# Characterization of Chemical Defensive Behavior and Associated Glands in the Destructive Invasive Longhorn Beetle *Aromia bungii*

**DOI:** 10.3390/insects17010089

**Published:** 2026-01-13

**Authors:** Ruixu Chen, Lisheng Hong, Jie Gao, Wenbo Wang, Quanmin Wen, Guangyu Wang, Tong Zhang, Tian Xu

**Affiliations:** 1 School of Landscape Architecture, Jiangsu Vocational College of Agriculture and Forestry, Zhenjiang 212499, China; 13735178534@163.com; 2 Co-Innovation Center for the Sustainable Forestry in Southern China, College of Forestry and Grassland, Nanjing Forestry University, Nanjing 210037, China; wbwang@njfu.edu.cn (W.W.); wqm@njfu.edu.cn (Q.W.); 19861835148@163.com (T.Z.); 3 School of Life Science, Institute of Life Science and Green Development, Hebei University, Baoding 071002, China; meigle24@163.com (J.G.); 17863800784@163.com (G.W.)

**Keywords:** *Aromia bungii*, chemical defense, defensive gland, longhorn beetle, invasive species

## Abstract

This study characterizes the chemical defense system of the invasive longhorn beetle *Aromia bungii*, a destructive pest of *Prunus* trees, addressing the limited understanding of chemical defensive mechanisms in Cerambycidae. High-speed cameras, environmental scanning electron microscopy (ESEM), dissection, and micro-CT imaging were used to investigate defensive behavior, and the structure of the defense system, in this beetle. Both sexes of *A. bungii* possess a pair of triangular, sac-like defensive glands symmetrically located in the metathorax, attached to the metasternum. Upon mechanical stimulation, white liquid defensive substances are rapidly ejected through a pair of slit-shaped openings (~200 µm) at the metasternum corners, without gland eversion, reaching over 50 cm. The average weight of substances ejected in first sprays was 7.95 ± 0.79 mg for females and 8.62 ± 2.13 mg for males (mean ± se), with no significant difference between sexes. However, the weight in second sprays after 10 days was significantly lower, at 2.93 ± 0.54 mg for females and 2.22 ± 0.40 mg for males (mean ± se), suggesting that the beetles cannot re-synthesize the substances soon after spray. The weight of ejected substances had no correlation with beetle body weight. Our findings represent the first detailed morphological and functional description of a chemical defense system in Cerambycidae, revealing a specialized metasternal gland and spray mechanism. The substantial but likely non-renewable defensive substances reflect an adaptive trade-off in energy allocation between reproduction and defense in this species that exhibits high fecundity but a short lifespan at the adult stage.

## Simple Summary

The red-necked longhorn beetle *Aromia bungii* is a destructive invasive pest that causes substantial damage to economically important stone fruit trees such as cherries and plums. While chemical defenses are common in insects, they are rarely reported and remain poorly understood in longhorn beetles (Cerambycidae). This study aimed to characterize the chemical defense system of this species for the first time. Using advanced imaging techniques, we identified a pair of unique, triangular-shaped, sac-like glands storing liquid defensive substances, located in the beetle’s metathorax. When threatened, the beetle rapidly ejects this liquid over a long distance through a pair of tiny openings on its body. A single spray contains a substantial amount of defensive substances. However, the beetle probably cannot quickly replenish these substances, because in a second spray performed after 10 days only a small amount was ejected. This indicates that the beetle may prioritize energy for reproduction over repeated syntheses of defensive substances. Understanding this highly specialized defense mechanism provides insights into this beetle’s survival and reproduction strategies which are valuable for developing novel strategies to manage this pest.

## 1. Introduction

**Figure 1:**
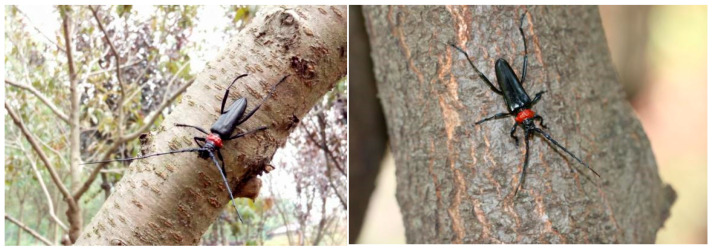
Field photograph of *Aromia bungii* adults. Male (**left**) and female (**right**).

The red-necked longhorn beetle *Aromia bungii* (Faldermann, 1835) (Coleoptera: Cerambycidae) is a destructive wood-boring pest widely distributed in East Asia (China, Korea, Vietnam, and Mongolia) [1,2,3]. Since 2012, invasions of *A. bungii* into Germany, Italy, and Japan, along with its recurrent interception in cargo destined for the USA and the UK, have underscored the formidable threat posed by its widespread dissemination across international borders [1,4,5,6,7]. This invasive pest has inflicted substantial harms on trees in the genus *Prunus* (Figure 1), particularly economically significant stone fruits like cherries, peaches, and plums, both in its endemic regions and in newly invaded territories [8,9,10].

The life span of *A. bungii* typically ranges from 1 to 3 years, with a predominant duration of 2 years [11,12]. Taking a two-year life history as an example, the female adult lays her eggs in bark crevices after mating. The eggs subsequently hatch into larvae that feed from the bark into the cambium and then the deep xylem [13]. They overwinter as larvae and construct a pupal chamber in the deep xylem by the end of the second year [14]. From the pupal chamber, the mature larvae excavate a tunnel to make an exit hole before prepupation, and then return to the pupal chamber for pupation during the following spring [12,14]. In China, adults emerge between June and August and live for around 53 days. In Japan, populations in Japan differ slightly in terms of adult emergence and longevity, a phenomenon which might be caused by differences in seasonal phenology [12]. Adults of *A. bungii* do not feed, and are typically capable of reproducing soon after emergence [15]. The female can lay over 300 eggs during her life [16].

Chemical defenses based on the synthesis and release of toxic or irritating substances are common in insects; these include the venoms of ants and bees, the odor of stink bugs, and the stink glands of papilionid larvae [17,18,19]. In Coleoptera, the chemical defense behaviors of bombardier beetles (Carabidae, Brachininae) have been extensively studied in bionics [20,21]. Nevertheless, chemical defense behavior has barely been found in Cerambycidae [22,23]. In a previous study, we reported a defensive behavior in *A. bungii*. The adults spray white liquid with a pungent scent from lateral body sides under external stimulation, and rose oxide is one of the major components in the spray [24]. So far, only a few cerambycid species have been found to exhibit chemical defense behavior, all of which, including *A. bungii*, belong to the tribe Callichromatini (subfamily Cerambycinae) [24,25,26]. However, with regard to chemical defense in cerambycid species, the associated glands, the chemical composition, the ejection mechanism, and the defensive function are all poorly understood.

In this study, we identified the locations and structures of the glands storing defensive substances, as well as the openings for ejection on body walls, in adult *A. bungii*. We also measured the weight of the defensive substances in single ejections and analyzed the relationship of this weight to beetle body weight. The results will contribute to deepening our understanding of chemical defense in Cerambycidae, and of survival and reproduction strategies in *A. bungii*.

## 2. Materials and Methods

### 2.1. Insect Sources

*Aromia bungii* adults were collected in the field from *Prunus cerasifera*, *Cerasus yedoensis*, and *Cerasus serrulata* trees on the campus of Hebei University and the Military Academy Square Park in Baoding, Hebei Province, China, from 10 July to 27 July 2023. All beetles were gently moved into plastic cups using a cup lid, without hand contact, to avoid the spray of defensive substances [24]. Beetles were individually kept in transparent plastic cups (10 cm diameter × 9 cm height) and then brought back to the laboratory. Two dental cotton rolls, containing ddH_2_O and 10% (*w*/*v*) sucrose solution, respectively, were provided for each beetle every three days. Adults were kept in the laboratory (25 °C, L:D = 16:8) until use. Because all beetles were collected in the field, their ages and mating statuses were unknown. The adults were sexed by comparing antenna length to body length [27].

### 2.2. Spray Process Recording

Active adults that did not spray after collection were chosen for recording. A high-speed camera (Revealer GR220M, Hefei Zhongke Junda Vision Technology Co. Ltd., Hefei, China) with a SIGMA lens (105 mm F2.8 EX DG MACRO OS, Sigma Corporation, Kawasaki, Japan) was used. Revealer Motion Analysis (v1.0.8, Hefei Zhongke Junda Vision Technology Co., Ltd., Hefei, China) together with Revealer Camera Control (new.6998c76d.20230615, Hefei Zhongke Junda Vision Technology Co. Ltd., Hefei, China) was used for controlling the camera and analyzing the images. The frames per second (FPS) was set at 1000, and the phase alternating line (PAL) was set at 25. In order to prevent heat from the light causing the beetles to become agitated, a box containing an ice pack and covered with black cloth was placed under the beetle. A dental cotton roll containing 10% (*w*/*v*) sucrose solution was provided to the beetle for holding. When recording, forceps were used to laterally compress the bases of the elytra or to compress the pronotum and sternum simultaneously.

### 2.3. Environmental Scanning Electron Microscopy

Microstructures of gland openings in 23 adults, including 13 females and 10 males, were scanned using environmental scanning electron microscopy (ESEM) (Quanta 200, FEI Company, Eindhoven, The Netherlands). The dissected glands were first ultrasonically cleaned in 75% ethanol and then washed with saline, followed by soaking in 70% FAA fixative solution. The specimens were air-dried for a few minutes and visualized at 200 V–30 kV.

### 2.4. Gland Dissection

A total of 20 beetles were killed using ethyl acetate. Killed beetles were individually placed on a Petri dish (90 mm diameter and 15 mm height). The wings and terga were removed to expose the internal structures of the thorax. Then, the thorax was immersed in 1% sodium hydroxide solution and boiled for 1 min to remove muscles and digestive structures, and to expose defensive glands. Saline was used to wash out the decomposed muscle tissue residue.

### 2.5. Micro-CT Imaging of the Defensive Gland

Cotton balls soaked with ethyl acetate were placed into the rearing container to kill beetles that had sprayed once. From 15–20 min until the beetles died, the legs and elytra were detached from the body. Saturated aqueous iodine solution was injected into each beetle body from the glandular opening via a 1 mL syringe.

Each specimen was pinned using a minute pin, attached to a 5 mL transparent centrifuge cube, and fixed with a small tissue ball. The scanner was a Bruker Skyscan1172 (Bruker Corporation, Billerica, MA, USA) with a SHT 11Mp camera. The camera pixel size was 9 µm, and the image pixel size ranged from 3.93~6.89 µm. The object-to-source distance was 93.430~263.5 mm, and the camera-to-source distance was 213.971~343.961 mm. The vertical object position was 30~32.468 mm. Source voltage and current were 74 kV and 131 uA, respectively. Camera binning was set to 1 × 1. In order to better differentiate the structures of low-density soft tissues within specimens, two different exposure times, 400 ms and 1520 ms, were used. The angular step was 0.4 degrees, and a total of 500 files with four frames per file were generated, resulting in total scan times from 42 m 24 s to 1 h 14 m 56 s. Files were reconstructed using the program NRecon (Version: 1.7.4.6), and the engine used was NReconServer (Version: 1.7.4.6). Beam hardening correction was set to 18%, compensating for the artifacts caused by the preferential absorption of low-energy X-rays. The 3D models were constructed via CTvox (version 3.3). Virtual section stacks in the three principal planes (coronal, sagittal, and axial) were exported in JPG format.

### 2.6. Weights of Defensive Substances Ejected

A total of 73 active beetles (27 male and 46 female) that did not spray after collection were chosen. A single beetle together with its rearing container was first weighed using a ten-thousandth balance (Sartorius BCA224i-1OCN, Sartorius AG, Göttingen, Germany). The beetle was then gently lifted without spray, and the rest weight was recorded. The difference between the two weights was recorded as the body weight of a single beetle.

The weight of the defensive substances ejected once by a beetle was measured following a previously reported method [24]. First, an empty 20 mL glass vial was weighed. Then, a beetle was introduced into the vial, and the bases of its elytra were compressed by using forceps. The vial cap was tightened immediately after the beetle was removed from the vial. The vial weight was measured again. The difference between the two weights was recorded as the weight of the defensive substances in the first ejection by a beetle. After ten days, the 30 adult beetles (13 male and 17 female) that were still alive were treated again, following the above steps. The weight of the substances in the second ejection by each beetle was recorded. Although it was unclear whether the beetles had previously sprayed defensive substances before collection, for simplicity, the two ejections performed in the laboratory were termed “the first spray” and “the second spray”.

### 2.7. Statistical Analysis

Because the data of the weights of ejected defensive substances failed to follow a normal distribution (Shapiro–Wilk test), non-parametric *t*-tests (Mann–Whitney *U* test) were used to compare weights between sexes and between first and second sprays. A Spearman correlation analysis was performed to test for correlation between the weights of ejected defensive substances and the weights of beetles. The analyses were performed using Microsoft Excel (Microsoft Office 365, released 2017. Redmond, WA, USA), SPSS (IBM SPSS Statistics 23) and GraphPad Prism 8 (GraphPad Software, San Diego, CA, USA).

## 3. Results

### 3.1. Spray Behavior

**Figure 2:**
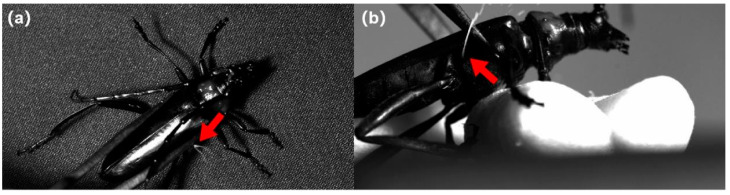
Vertical view (**a**) and side view (**b**) of spray of white liquid defensive substances performed by a female *Aromia bungii.* The red arrows point to the sites from where the white defensive substances were sprayed.

When compressed, the beetles sprayed white liquid substances directly from the sides near the coxal fossa of the hind legs on the metathorax, without extending any specific glands (Figure 2a,b). The two sides can spray separately (Figure 2a). This behavior can be performed rapidly: more than once within 2 s. Observation showed that the radius of the spray can be over 50 cm. A video of full spray behavior can be found in the Supporting Information.

### 3.2. The Gland Opening

**Figure 3:**
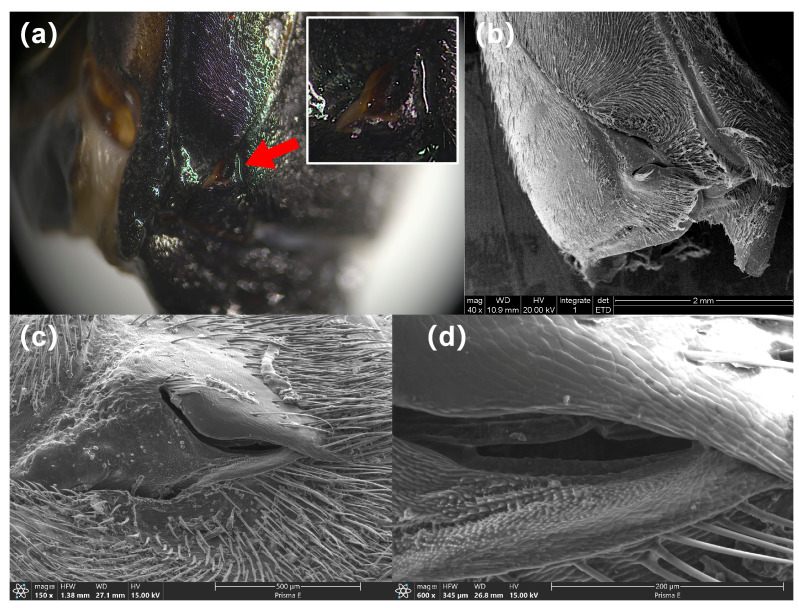
Structure of the gland opening. The red arrow points to the hole through which the white defensive substances were sprayed. Gland opening under light microscopy (**a**) and under environmental scanning electron microscopy (**b**–**d**).

The gland opening on each side of the thorax was a slit, narrowly “V” shaped with a diameter of about 1 mm, located at the corner of the metastethium (Figure 3a). ESEM scanning showed that the body wall at the V-shaped opening is invaginated inwards (Figure 3b). At the base of this depression, a separate slit-like opening, approximately 200 µm in length, is present (Figure 3c,d).

### 3.3. Defensive Gland

**Figure 4:**
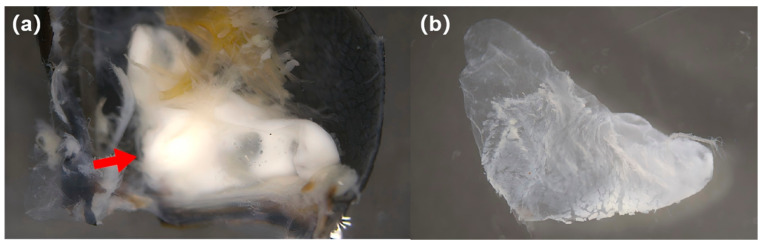
(**a**) A gland containing white defensive substances attached to the inner surface of the metastethium; and (**b**) a gland without defensive substances. The red arrow points to the gland containing white defensive substances.

An internal gland was found to connect to the opening. The gland was filled with white defensive substances (Figure 4a). The defensive gland was attached to the inner surface of the metastethium through a groove. In general, the shape of the gland was close to that of an isosceles triangle (Figure 4b) with a long side of about 140–190 mm.

**Figure 5:**
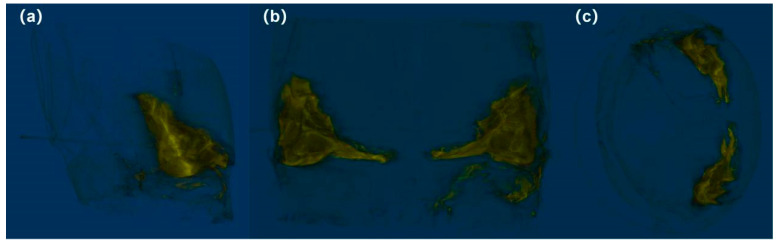
Three-dimensional reconstruction diagram of the gland. The side view (**a**), vertical view (**b**), and front view (**c**) of the gland. The front view was taken at the front of the beetle’s metastethium.

A three-dimensional diagram of the defensive gland was reconstructed using Micro-CT (Figure 5), in which a yellow area represented the defensive gland filled with iodine solution. Iodine solution was injected through the opening at the corner of metastethium, indicating that the opening and the gland were connected. The yellow area on the lower right side of the vertical view (Figure 5b) was caused by iodine fluid that seeped from the injection site and stained the muscles during the injection process.

When using the reconstruction image for three-dimensional imaging, some of the opacity of the body wall was retained in images as a black shadow which could be used to determine the location of the gland in the thorax. The results showed that the glands formed a pair, each triangular in shape, symmetrically distributed in the metathorax close to the metastethium. The irregular folds observed on the surface of the structure were due to the fact that the iodine solution did not fully expand the soft gland wall. When the gland fully expanded in physiological saline solution, its outline was more regular, as can be seen in Figure 4.

### 3.4. Weights of Defensive Substances Ejected

**Figure 6:**
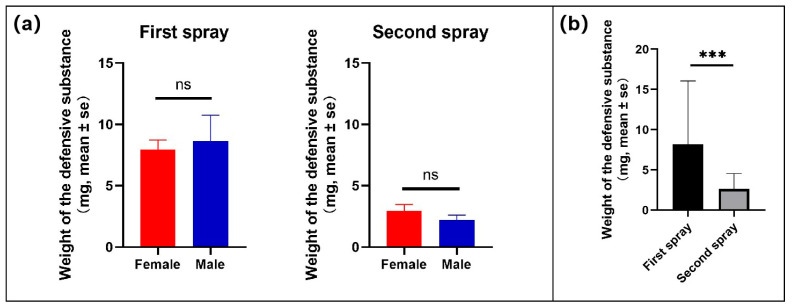
Weights of defensive substances ejected in first and second sprays by females and males (**a**), and a comparison between weights of substances ejected in first and second sprays (**b**). Asterisks indicate significant differences between the weights of defensive substances in the first and second sprays (Mann–Whitney *U* test, *p* < 0.01); ns indicates no significant difference in the weights of defensive substances between sexes (Mann–Whitney *U* test, *p* > 0.05).

The weights of defensive substances in the first and second sprays both showed no significant differences between males and females (Mann–Whitney *U* test: first spray, *p* = 0.7278; second spray, *p* = 0.3260) (Figure 6a). In the first spray, the mean values of the substance weights were 7.95 ± 0.79 mg for females and 8.62 ± 2.13 mg for males (mean ± se). In the second spray, the mean values of the substance weights were 2.93 ± 0.54 mg for females and 2.22 ± 0.40 mg for males (mean ± se). The substance weights in the first spray were significantly greater than those in the second spray (Mann–Whitney *U* test: *p* < 0.05) (Figure 6b). The weights of the ejected substances had no significant correlation with beetle weights (Spearman correlation analysis: *p* = 0.088).

## 4. Discussion

In the present study, we show the full process of chemical defense behavior, and describe related physical structures—including the gland storing defensive substances, and its opening—in *Aromia bungii*, this being, as far as we know, the first such detailed description for a Cerambycidae beetle. In *A. bungii*, the defensive glands are a pair of triangular, sac-like structures situated adjacent to the metasternum. Upon stimulation, these glands eject defensive substances through slit-shaped openings located on either side of the metasternum. Amounts of substances ejected in a single spray were found to be substantial (approximately 8.3 mg on average), but these amounts were much lower in second sprays after 10 days.

Chemical defense behavior has been widely found in insects across various orders, including Blattodea, Dermaptera, Phasmatodea, Hemiptera, Lepidoptera, Orthoptera, and Coleoptera, with defensive compounds shown to be stored in specific structures in the integuments, such as the cuticular cavities, subcuticular compartments, or exocrine defense glands [28,29,30,31,32]. Some studies have reported the existence of metasternal glands and mandibular glands in some cerambycid species; these glands may be used to produce defensive secretions, but detailed morphological and locational descriptions are lacking [23,26,30,33,34]. In Coleoptera, chrysomeline larvae have nine pairs of dorsally located exocrine defense glands which are composed of secretory cells, canal cells, and a chitin-coated reservoir. Upon attack, the larvae contract specific muscles to extend the glandular reservoirs and release droplets of defensive secretions onto their backs [35,36]. In the family Carabidae, the defensive chemicals are stored in glands located in the abdomen, and they are rapidly sprayed from the abdominal tip with the ability to flexibly adjust the direction [37]. Differently, the defensive substances of *A. bungii* are stored in a pair of sac-like glands attached to the metasternum, and they are sprayed directly through the holes, without an eversion of specific sac. Compared with defensive glands in other coleopterans, which are usually located in the abdomen, the metasternal glands of *A. bungii* may be advantageous for a more rapid response to threaten predators such as birds without the need to adjust abdominal posture. Notably, this gland location and this method of chemical release are very similar to those of some species in Phasmatodea [38]. In *Anisomorpha buprestoides*, the defensive substances are ejected from openings located on the metathorax following stimulation, reaching a distance of approximately half a meter [39,40]. Moreover, in the musk beetle *Aromia moschata*, a congener of *A. bungii*, the defensive substances are also sprayed from the thorax and have a chemical composition similar to that of *A. bungii* [24]. Because the few cerambycid species that have been found to exhibit chemical defense behavior have all belonged to the tribe Callichromatini [24,25,26], we thus hypothesize that these species and possibly others in the tribe Callichromini may also possess similar metasternal defensive glands and spray mechanism to that found in *A. bungii*.

According to the classification of exocrine secretory release behaviors by Foster and Casas (2025), the rapid, long-distance (>50 cm) ejection by *A. bungii* upon mechanical stimulation can be defined as a “Spray” [41]. Based on the dissections of *A. bungii* adults, we speculate that the spray mechanism in this beetle may involve inflation of a muscle-sheathed air sac in the thorax upon physical stimulation. This inflation would exert pressure on the elastic sac-like gland that contains the defensive substances, forcing the white liquid to be expelled through the holes. This passive pressure-driven mechanism is common in the chemical defense systems of many insects, as it enables a rapid response without the need for complex muscular control [42]. Future research employing high-precision pressure sensors and microscopy techniques may be performed to measure changes in internal glandular pressure during the spray process, elucidating the underlying mechanism.

In the present study, we found that the amount of defensive substances in the first spray was large (approx. 8 mg), but the quantity of chemicals ejected on the second occasion (10 days after the first spray) was significantly lower. This result suggests that the defensive substances cannot be re-synthesized soon after spray, possibly due to a high synthesis cost [43]. At the adult stage, *A. bungii* do not feed and have a short life span but high fecundity. Thus, the substances may be able to compensate for the adults’ means of predator avoidance, and most of the energy is probably allocated to reproduction [44]. Furthermore, because all the beetles used in the study were field-collected, we are still unclear about the stage at which the gland and the defensive substances are generated. It seems that there is no need for larvae living deeply inside trees to develop such a defensive strategy. We speculate that the gland may be formed during the pupal stage, and that the chemicals may be synthesized simultaneously or soon after eclosion; however, these are matters that need further investigation.

In conclusion, this study elucidates a highly specialized chemical defense system in the destructive invasive longhorn beetle *A. bungii*. Such a defensive strategy may be adaptive for the need of a trade-off between survival and reproduction in this species. The findings may deepen our understanding of the chemical defense behavior in Cerambycidae.

## Data Availability

The original contributions presented in this study are included in the article/Supplementary Material. Further inquiries can be directed to the corresponding authors.

## Supplementary Materials

The following supporting information can be downloaded at: https://www.mdpi.com/article/10.3390/insects17010089/s1, Video S1: Spray process recorded by high-speed camera.
